# H1N1‐Related Hemophagocytic Lymphohistiocytosis Complicated by Severe Rhabdomyolysis in a Pediatric Patient: A First Case Report

**DOI:** 10.1155/crpe/3078496

**Published:** 2026-03-13

**Authors:** Lucia Marseglia, Antonella Gambadauro, Federica Xerra, Annalisa Cacciola, Immacolata Rulli, Valeria Chirico, Raffaella Mallamace, Eloisa Gitto

**Affiliations:** ^1^ Neonatal and Pediatric Intensive Care Unit, Department of Human Pathology in Adult and Developmental Age “Gaetano Barresi”, University Hospital “G. Martino”, Messina, Italy, polime.it; ^2^ Pediatric Nephrology and Dialysis Unit, Department of Human Pathology in Adult and Developmental Age “Gaetano Barresi”, University Hospital “G. Martino”, Messina, Italy, polime.it

**Keywords:** H1N1, hemophagocytic lymphohistiocytosis, HLH, influenza, intensive care unit, pediatric, rhabdomyolysis

## Abstract

**Background:**

Hemophagocytic lymphohistiocytosis (HLH) is a rare and often life‐threatening hyperinflammatory disorder, mainly caused by infections, malignancies, or autoimmune diseases. Influenza A (H1N1) has occasionally been linked to pediatric HLH cases. Rhabdomyolysis, relatively uncommon in children, can be a complication of viral infections, including influenza. To our knowledge, this is the first reported pediatric case where H1N1 infection was complicated by both HLH and severe rhabdomyolysis.

**Case Presentation:**

We describe the case of a previously healthy 4‐year‐old girl who was hospitalized with high fever, gastrointestinal symptoms, and marked somnolence. Blood tests revealed severe rhabdomyolysis (creatine phosphokinase [CPK] > 100,000 U/L) and metabolic acidosis. Influenza A (H1N1) infection was confirmed by a nasopharyngeal swab. Despite prompt initiation of antiviral and supportive therapies, the patient developed persistent fever, pancytopenia, hyperferritinemia, hypertriglyceridemia, hepatosplenomegaly, and cardiac involvement. Bone marrow biopsy confirmed hemophagocytosis, according to HLH‐2004 criteria. She was treated with high‐dose methylprednisolone and intravenous immunoglobulin (IVIG), with rapid clinical improvement. The patient was discharged after 16 days without sequelae.

**Conclusions:**

This case describes a rare but severe presentation of Influenza A infection in a child, complicated by both HLH and severe rhabdomyolysis. The combination of a hyperinflammatory condition and multiorgan dysfunction highlights the importance of early recognition and multidisciplinary management. Our case is the first documented pediatric case of H1N1‐related HLH with severe rhabdomyolysis in pediatric populations, and a review of the literature confirms the rarity of this association. Clinicians should maintain a high index of suspicion for HLH in children with persistent fever, cytopenia, hyperferritinemia, and organ involvement during viral infections. The potential coexistence of rhabdomyolysis should be considered in the presence of persistent high CPK levels to prevent serious complications such as acute kidney injury.

## 1. Introduction

Hemophagocytic lymphohistiocytosis (HLH) is a life‐threatening hyperinflammatory syndrome characterized by a “cytokine storm” and the pathologic accumulation of activated T‐lymphocytes and macrophages in various organs [1–3]. This process can lead to multiorgan failure (MOF) and high mortality if not promptly recognized [4]. In pediatric patients, diagnosis is typically established using the HLH‐2004 criteria, requiring the fulfillment of at least five out of eight clinical and laboratory parameters [5, 6]. HLH is traditionally classified into primary (genetic) and secondary (acquired) forms. Secondary HLH is more prevalent and can be triggered by malignancies, autoimmune disorders, or infections (HLH‐I), with viral pathogens being the most frequent triggers in the pediatric population [7–9]. While various viral agents are well‐documented, Influenza A (H1N1) has been only rarely described as a potential trigger of HLH in children [10–15]. Interestingly, the postcoronavirus disease‐19 (COVID‐19) era has seen a shift in the severity of common viral infections, including H1N1. This has been attributed to the “immunity debt” resulting from reduced pathogen exposure during pandemic‐related social distancing [16]. While the primary severe complications of H1N1 are respiratory, rare systemic manifestations such as rhabdomyolysis have been reported. Herein, we describe a unique pediatric case of influenza H1N1 infection complicated by both rhabdomyolysis and subsequent HLH‐I, highlighting the diagnostic challenges in the current epidemiological landscape.

## 2. Case Presentation

### 2.1. Clinical Presentation

A 4‐year‐old African female was admitted to the pediatric intensive care unit (PICU) of the University Hospital of Messina (Italy) in November 2024. She was a previously healthy child, born prematurely from a twin pregnancy, with appropriate growth and psychomotor development. She had a regular vaccine schedule and no significant past medical history. The day before hospital admission, she developed high fever (40.5°C), cough, vomiting, and diarrhea, with poor food intake, followed by marked somnolence and tachypnea. On admission, she was somnolent and irritable (BT: 38.5°C, HR: 145 bpm, RR: 43 bpm, SpO2: 90%, and BP: 118/80 mmHg). Physical examination showed a hyperemic pharynx and tonsils; chest, cardiac, and skin examinations were unremarkable. A broad‐spectrum antibiotic (ceftazidime) was started by considering the severe general status, without improvement.

### 2.2. Investigations and Diagnosis

Blood gas analysis showed metabolic acidosis (pH 7.24, HCO3:12.8 mmol/L, BE:15). Laboratory investigations revealed neutrophilic leukocytosis, increased C‐reactive protein (CRP), and severe rhabdomyolysis (Table 1). Initial imaging (chest radiograph, echocardiogram, and abdominal ultrasonography) and neurological workup (brain CT and lumbar puncture) were normal. A respiratory microbiological panel (RT‐PCR) on a nasal swab revealed Influenza A (H1N1‐2009). Despite initial improvement in muscle enzymes through aggressive hydration and supportive care, the patient developed persistent fever and clinical deterioration. Extensive screening for other infectious agents (EBV; CMV; HIV; parvovirus B19; toxoplasmosis; hepatitis A, B, and C; and blood/urine cultures) was negative. On the third day, new laboratory findings emerged: pancytopenia, elevated transaminases, LDH, triglycerides, and ferritin (> 10,000 ng/mL), alongside hypofibrinogenemia (Table 1). New‐onset hepatosplenomegaly and free abdominal fluid were detected via ultrasonography. Cardiac evaluation revealed mild multivalvular insufficiency (aortic, mitral, and tricuspid), coronary hyperechogenicity, and preserved ejection fraction, associated with increased troponin and BNP levels. Due to the clinical setting, sIL‐2R levels and NK cell activity were not assessed as the patient already met 6 out of 8 HLH‐2004 criteria. Bone marrow biopsy confirmed hemophagocytosis, establishing the diagnosis of HLH secondary to Influenza A H1N1.

**TABLE 1:** Laboratory parameters of our case.

Parameter	Day 1	Day 3	Day 5	Day 16	Reference range
Hb (g/dL)	12.0	9.6	8.9	10.1	11.0–15.0
Leukocytes (× 10^9^/L)	9.2	5.1	3.8	17	6.0–12.0
Platelets (× 10^9^/L)	60	43	41	499	150–350
CRP (mg/L)	152	85	80	1	0–0.5
ESR (mm/1^st^ hr)	NA	NA	12	NA	0–20
Ferritin (ng/mL)	NA	1640	1800	173	13–150
Fibrinogen (mg/dL)	446	NA	147	231	200–400
Triglycerides (mg/dL)	NA	316	353	NA	10–150
AST (IU/L)	3823	3767	2292	138	0–42
ALT (IU/L)	729	735	587	221	0–50
LDH (IU/L)	1627	4506	NA	667	135–225
CPK (IU/L)	68,643	119,785	22,503	2311	0–200

### 2.3. Treatment and Outcome

On the second day, oral oseltamivir was started. Following the HLH diagnosis, a combination of intravenous high‐dose methylprednisolone (30 mg/kg/day for 3 days) and intravenous immunoglobulin (IVIG) (2 g/kg over 5 days) was initiated. The corticosteroid dose was then gradually tapered over the following four weeks. Etoposide was withheld due to the rapid clinical response to first‐line therapy and the secondary nature of the syndrome. After the second dose of immunosuppressive therapy, the patient became afebrile with progressive normalization of all parameters. She was discharged after 16 days in stable condition. During follow‐up, autoimmune and rheumatological conditions were excluded.

## 3. Discussion

We describe a rare and complex case of a 4‐year‐old female who developed a hyperinflammatory syndrome with features suggestive of secondary HLH and severe rhabdomyolysis during Influenza A (H1N1) infection. Her case underscores the diagnostic and therapeutic challenges posed by overlapping hyperinflammatory syndromes in pediatric patients and the potential for severe multisystem involvement triggered by common respiratory pathogens. In our patient, the diagnosis of HLH secondary to Influenza A (H1N1) was confirmed by the fulfillment of six out of eight HLH‐2004 criteria [6]. Specifically, the clinical course was characterized by persistent high‐grade fever, splenomegaly, pancytopenia, hyperferritinemia (> 10,000 ng/mL), hypertriglyceridemia, and biopsy‐proven hemophagocytosis. While H1N1 is a well‐documented cause of respiratory failure, its role as a trigger for the “cytokine storm” typical of secondary HLH is increasingly recognized in the pediatric population [11–15]. Our case reinforces this association, suggesting that H1N1 should be promptly considered as a potential driver of immune dysregulation when standard supportive care fails to stabilize the patient.

The association between Influenza A infection and HLH has been previously documented in a limited number of pediatric case reports (see Tables 2(a) and 2(b)). Shrestha et al. [14] described a fatal case of a previously healthy 8‐year‐old girl who succumbed to fulminant HLH following H1N1 influenza infection, despite intensive supportive care. Curiously, this patient was the only one to not receive oseltamivir in the case reports described in the literature (Tables 2(a) and 2(b)). Probably the adoption of immunosuppressive treatment (IVIG and steroids) without the administration of antiviral treatment, along with influenza infection complicated by HLH, may not be sufficient in rapidly progressive and fatal cases. Conversely, Vignesh et al. [12] described a severe case of an infant of 6 months who developed HLH during Influenza A infection with severe hyperferritinemia and pancytopenia, requiring immunosuppressive treatment and oseltamivir. This child, despite the difficult course of the disease requiring intubation and mechanical ventilation, responded to treatment and was discharged without sequelae [12]. These cases illustrate the rapid progression and severity of HLH when associated with Influenza A and the need for early therapeutic intervention.

**TABLE TABLE​ 2:** Care reports of pediatric patients with HLH secondary to Influenza A (H1N1) extracted from literature search.

Id	1	2	3	4	5
Reference	[11]	[12]	[13]	[14]	[15]
Publication (year)	2010	2022	2011	2015	2013
Gender	F	M	M	F	M
Age	17 years	6 months	2 months	8 years	4 years
Comorbidity	No	Omen syndrome	No	No	No
Symptoms presentation	Asthenia, pneumonia, myalgia, high fever	High fever and persistent cough	High fever, malaise, upper respiratory symptoms	High fever, colicky abdominal pain, rhinorrhea, cough	Fever, malaise, myalgia, cough, respiratory distress, oliguria
H1N1 detected	Yes	Yes	Yes	Yes	Yes
Hb (g/dL)	7.8	9.8	8.9	8.5	9.2
Leukocytes (× 10^9^/L)	0.5	4.2	1.2	2.9	3
Platelets (× 10^9^/L)	28	150	40	29	56
CRP (mg/L)	214	79	8	15	19
ESR (mm/1^st^ hr)	NA	NA	12	15	NA
Ferritin (ng/mL)	1500	> 2000	10,423	12,000	875
Fibrinogen (mg/dL)	NA	46	111	NA	120
Triglycerides (mg/dL)	235	255	189	390	268
AST (IU/L)	52	133	756	280	208
ALT (IU/L)	23	116	117	150	72
LDH (IU/L)	1657	NA	2110	NA	893
Fever	Yes	Yes	Yes	Yes	Yes
Respiratory symptoms	Pneumonia	Bilateral crepitations and retractions	Dry cough and rhinorrhea	Cough and rhinorrhea; then distress	Cough, respiratory distress
Lung imaging	Interstitial pneumonia (CT)	NA	NA	NA	Infiltrates in both lung fields and bilateral pleural effusions (XR)
Neurological symptoms	No	No	No	Generalized tonic‐clonic seizures	No
Brain imaging	NA	NA	NA	NA	NA
Skin‐mucosae	NO	Erythematous macular rash	NO	Multiple purpuric eruptions	Diffuse petechial eruption
Splenomegaly	Yes	Yes	Yes	Yes	Yes
Hepatomegaly	Yes	Yes	Yes	Yes	Yes
GI symptoms	Severe jaundice	NO	NO	Abdominal pain, jaundice, gallbladder wall edema, ascites	No
Rhabdomyolysis	No	No	No	No	No
Bone marrow	Hemophagocytosis	Hemophagocytosis	Hemophagocytosis	Hemophagocytosis	Hemophagocytosis
Antibiotics	Imipenem/cilastatin	Broad spectrum	Cefepime	NO	Meropenem
Antivirals	Oseltamivir	Oseltamivir	Oseltamivir	NO	Oseltamivir
Corticosteroids	Prednisone (1 mg/kg/day)	Yes	No	Methylprednisolone (30 mg/kg/dose)	No
IVIG	No	Yes	No	Yes	Yes
Blood transfusion	No	Yes	No	No	No
Ventilatory support	NIV and COT by face mask	Yes	No	MV	No
LOS (day)	21	NA	NA	NA	14
Co‐infection	No	*K. pneumoniae*	No	No	No
Sequelae	No	No	No	MOF	No
Death	No	No	No	Yes	No

*Note:* 2A: demographics, infection, and laboratory findings. 2B: clinical course, therapy, and outcomes. ALT, alanine aminotransferase; AST = aspartate aminotransferase; GI = gastrointestinal; Hb = hemoglobin; HLH = hemophagocytic lymphohistiocytosis; IVIG = intravenous immunoglobulin; LDH = lactate dehydrogenase.

Abbreviations: COT, conventional oxygen therapy; CRP, C‐reactive protein; CT, computed tomography; ESR, erythrocyte sedimentation rate; ICU, intensive care unit; LOS, length of stay; MOF, multiple organ failure; MV, mechanical ventilation; NA, not available; NIV, noninvasive ventilation; XR, X‐ray.

Our patient’s clinical course was complicated by the development of rhabdomyolysis, with a peak of creatine phosphokinase (CPK) level equal to 148,000 U/L in the first days of admission. Rhabdomyolysis is a rare complication of influenza infection, and it was documented also in the pediatric population [17]. It can result from direct viral invasion of muscle tissue, immune‐mediated muscle injury, or systemic effects of hyperinflammation [18]. In our patient, the combination of severe muscle damage and hyperinflammatory syndrome may have created a vicious cycle of worsening systemic inflammation and multiorgan dysfunction. Few reports have described the co‐occurrence of HLH and rhabdomyolysis in pediatric patients [19]. Furthermore, to our knowledge, the presence of both conditions (HLH and rhabdomyolysis) was never described in the literature during Influenza A infection. A report described their occurrence in an adult after inactivated influenza vaccination [20]. The simultaneous presence of these conditions significantly increases the risk of acute kidney injury, fluid and electrolyte imbalances, and cardiovascular compromise [19]. In our case, the therapeutic management included aggressive intravenous hydration, urine alkalinization, correction of electrolyte alterations, and diuretic therapy, which contributed to the prevention of dialysis‐requiring acute kidney injury. The therapeutic approach in our child fulfilled the HLH management indications. High‐dose IVIG and corticosteroids (methylprednisolone) were administered early, based on the hypothesis of HLH. Additional supportive therapies included low‐dose heparin, inotropic support, and plasma/albumin infusions to treat hypoalbuminemia and maintain oncotic balance. The clinical and laboratory improvement observed following initiation of immunosuppressive therapy supports the utility of early intervention in cases of suspected HLH.

The neurological, cardiological and gastrointestinal symptoms observed in our patient (initial somnolence, irritability, multivalvular insufficiency, and the presence of free abdominal fluid) highlight the multisystem nature of the inflammatory process. The clinical presentation of our patient needed a full neurologic workup to rule out CNS infection or neuroinflammatory involvement, as both are recognized complications in HLH. However, neuroimaging and cerebrospinal fluid studies were normal. Gastrointestinal involvement, including abdominal pain and ascites, has also been reported in HLH and should be carefully monitored and treated [14]. Cardiac involvement was not described in other reports on HLH secondary to Influenza A (Table 2). However, it is commonly described in other hyperinflammatory syndromes of infancy, such as Kawasaki disease and multisystem inflammatory syndrome in children (MIS‐C), suggesting a possible overlapping of these conditions [21–23].

This case contributes to the growing literature on the intersection between severe viral infections and hyperinflammatory syndromes in children. It emphasizes the importance of considering HLH in children with persistent fever, pancytopenia, hyperferritinemia, and multiorgan involvement, especially during Influenza A infection. The co‐occurrence of rhabdomyolysis further complicates management and necessitates aggressive supportive care.

## 4. Conclusions

In the post‐COVID‐19 era, the clinical spectrum of common respiratory viruses such as Influenza A (H1N1) appears to be evolving, with a potential for more severe hyperinflammatory presentations. This case demonstrates that H1N1 can trigger a rapid progression toward both severe rhabdomyolysis and secondary HLH in the pediatric population. Maintaining a high index of suspicion is crucial, as the overlapping symptoms of viral infection and HLH can delay diagnosis. Our findings suggest that early intervention with high‐dose corticosteroids and IVIG can be effective in stabilizing immune dysregulation, potentially avoiding more intensive chemotherapy. Further study is warranted to better understand the pathophysiological link between viral‐induced muscle injury and the subsequent cytokine storm to optimize treatment algorithms for pediatric hyperinflammatory syndromes.

**Nomenclature**
ALT: Alanine aminotransferase
AST: Aspartate aminotransferase
BE: Base excess
BNP: B‐type natriuretic peptide
CD25: Soluble interleukin‐2 receptor *α*
CK/CPK: Creatine phosphokinase
CMV: Cytomegalovirus
COVID‐19: Coronavirus disease 2019
CRP: C‐reactive protein
CT: Computed tomography
EBV: Epstein−Barr virus
ESR: Erythrocyte sedimentation rate
GI: Gastrointestinal
Hb: Hemoglobin
HHV‐6: Human herpesvirus type 6
HHV‐8: Human herpesvirus type 8
HIV: Human immunodeficiency virus
HLH: Hemophagocytic lymphohistiocytosis
HLH‐I: Infection‐associated hemophagocytic lymphohistiocytosis
HLH‐T: Tumor‐associated hemophagocytic lymphohistiocytosis
H1N1: Influenza A virus subtype H1N1
ICU: Intensive care unit
IL: Interleukin
IVIG: Intravenous immunoglobulin
LDH: Lactate dehydrogenase
LOS: Length of stay
MAS: Macrophage activation syndrome
MIS‐C: Multisystem inflammatory syndrome in children
MOF: Multiple organ failure
MRI: Magnetic resonance imaging (if mentioned; not sure, please confirm)
MV: Mechanical ventilation
NK: Natural killer (cells)
NIV: Noninvasive ventilation
PCR: Polymerase chain reaction
PICU: Pediatric intensive care unit
RT‐PCR: Reverse transcription polymerase chain reaction
SARS‐CoV‐2: Severe acute respiratory syndrome coronavirus 2
sIL‐2R: Soluble interleukin‐2 receptor
SpO_2_
: Peripheral oxygen saturation
XR: X‐ray

## Author Contributions

Conceptualization, supervision, and validation: Lucia Marseglia and Eloisa Gitto. Data curation, formal analysis, investigation, methodology, and writing−original draft: Antonella Gambadauro and Federica Xerra. Writing– review and editing: Lucia Marseglia, Annalisa Cacciola, Valeria Chirico, and Raffaella Mallamace .

## Funding

This research received no external funding.

Open access publishing facilitated by Universita degli Studi di Messina, as part of the Wiley ‐ CRUI‐CARE agreement.

## Disclosure

All authors read and approved the final manuscript.

## Ethics Statement

This study was conducted in accordance with the Declaration of Helsinki. Written informed consent was obtained from the patient’s parents.

## Consent

Please see the Ethics Statement.

## Conflicts of Interest

The authors declare no conflicts of interest.

## Supporting Information

Supporting Table provides a detailed overview of the HLH‐2004 diagnostic criteria used for the evaluation of the patient.

## Supporting information


**Supporting Information** Additional supporting information can be found online in the Supporting Information section.

## Data Availability

All data are available from the corresponding author on reasonable request.

## References

[1] Osugi Y. , Hara J. , Tagawa S. et al., Cytokine Production Regulating Th1 and Th2 Cytokines in Hemophagocytic Lymphohistiocytosis, Blood. (June 1997) 89, no. 11, 4100–4103, 10.1182/blood.v89.11.4100. 9166851

[2] Komp D. M. , McNamara J. , and Buckley P. , Elevated Soluble interleukin-2 Receptor in Childhood Hemophagocytic Histiocytic Syndromes, Blood. (June 1989) 73, no. 8, 2128–2132, 10.1182/blood.v73.8.2128.2128. 2786434

[3] Aricò M. , Danesino C. , Pende D. , and Moretta L. , Pathogenesis of Haemophagocytic Lymphohistiocytosis, British Journal of Haematology. (September 2001) 114, no. 4, 761–769, 10.1046/j.1365-2141.2001.02936.x, 2-s2.0-0034795745. 11564062

[4] Tang Y. , Xu X. , Song H. et al., Early Diagnostic and Prognostic Significance of a Specific Th1/Th2 Cytokine Pattern in Children With Haemophagocytic Syndrome, British Journal of Haematology. (October 2008) 143, no. 1, 84–91, 10.1111/j.1365-2141.2008.07298.x, 2-s2.0-51249099119. 18673367

[5] Henter J. , Horne A. , Aricó M. et al., HLH‐2004: Diagnostic and Therapeutic Guidelines for Hemophagocytic Lymphohistiocytosis, Pediatric Blood and Cancer. (February 2007) 48, no. 2, 124–131, 10.1002/pbc.21039, 2-s2.0-33845619137. 16937360

[6] Hines M. R. , von Bahr Greenwood T. , Beutel G. et al., Consensus-Based Guidelines for the Recognition, Diagnosis, and Management of Hemophagocytic Lymphohistiocytosis in Critically Ill Children and Adults, Critical Care Medicine. (May 2022) 50, no. 5, 860–872, 10.1097/ccm.0000000000005361. 34605776

[7] Ishii E. , Ohga S. , Imashuku S. et al., Nationwide Survey of Hemophagocytic Lymphohistiocytosis in Japan, International Journal of Hematology. (July 2007) 86, no. 1, 58–65, 10.1532/ijh97.07012, 2-s2.0-34547633673. 17675268

[8] Fisman D. N. , Hemophagocytic Syndromes and Infection, Emerging Infectious Diseases. (December 2000) 6, no. 6, 601–608, 10.3201/eid0606.000608, 2-s2.0-0034486618. PMC2640913 11076718

[9] Mostaza-Fernández J. L. , Guerra Laso J. , Carriedo Ule D. , and Ruiz de Morales J. M. G. , Hemophagocytic Lymphohistiocytosis Associated With Viral Infections: Diagnostic Challenge and Therapeutic Dilemma, Revista Clínica Española. (August 2014) 214, no. 6, 320–327, 10.1016/j.rceng.2014.03.004. PMC7129636 24796638

[10] Maakaroun N. R. , Moanna A. , Jacob J. T. , and Albrecht H. , Viral Infections Associated With Haemophagocytic Syndrome, Reviews in Medical Virology. (March 2010) 20, no. 2, 93–105, 10.1002/rmv.638, 2-s2.0-77649155207. PMC7169125 20127750

[11] Zheng Y. , Yang Y. , Zhao W. , and Wang H. , Novel Swine-Origin Influenza A (H1N1) Virus-Associated Hemophagocytic Syndrome—A First Case Report, American Society of Tropical Medicine and Hygiene. (April 2010) 82, no. 4, 743–745, 10.4269/ajtmh.2010.09-0666, 2-s2.0-77950950216.

[12] Vignesh P. , Anjani G. , Kumrah R. et al., Features of Hemophagocytic Lymphohistiocytosis in Infants With Severe Combined Immunodeficiency: Our Experience From Chandigarh, North India, Frontiers in Immunology. (June 2022) 13, 10.3389/fimmu.2022.867753. PMC9260510 35812426

[13] Özdemir H. , Çiftçi E. , Eü İ. , Ertem M. , İnce E. , and Doğru Ü. , Hemophagocytic Lymphohistiocytosis Associated With 2009 Pandemic Influenza a (H1N1) Virus Infection, Journal of Pediatric Hematology. (March 2011) 33, no. 2, 135–137, 10.1097/mph.0b013e3181f46baf, 2-s2.0-79952154001. 21285906

[14] Shrestha B. , Omran A. , Rong P. , and Wang W. , Report of a Fatal Pediatric Case of Hemophagocytic Lymphohistiocytosis Associated With Pandemic Influenza A (H1N1) Infection in 2009, Pediatrics and Neonatology. (June 2015) 56, no. 3, 189–192, 10.1016/j.pedneo.2013.03.006, 2-s2.0-84931562581. 23602384

[15] Demircioğlu F. , Kazancı E. , Genç D. B. , Erdoğan H. , Göksügür S. B. , and Bekdaş M. , H1N1 Infection-Related Hemophagocytic Lymphohistiocytosis in a Child, Turkish Journal of Hematology. (2013) 30, no. 4, 426–428, 10.4274/tjh.2013.0022. PMC3874979 24385840

[16] Liu H. F. , Hu X. Z. , Huang R. W. et al., Evaluation of Disease Severity and Prediction of Severe Cases in Children Hospitalized With Influenza A (H1N1) Infection During the Post-COVID-19 Era: A Multicenter Retrospective Study, BMC Pediatrics. (April 2024) 24, no. 1, 10.1186/s12887-024-04645-x. PMC10985932 38566022

[17] Ishiwada N. , Takada N. , Okunushi T. et al., Rhabdomyolysis Associated With Influenza A/H1N1 2009 Infection in a Pediatric Patient, Pediatrics International. (October 2012) 54, no. 5, 703–705, 10.1111/j.1442-200x.2012.03571.x, 2-s2.0-84866991546. 23005902

[18] Khan F. Y. , Rhabdomyolysis: A Review of the Literature, Netherlands Journal of Medicine. (October 2009) 67, no. 9, 272–283. 19841484

[19] Maloney L. T. , Baz B. , and Hazra D. , HLH-Like Syndrome and Rhabdomyolysis in an Adolescent Patient, Pediatrics. (November 2021) 148, no. 5, 10.1542/peds.2021-050162. 34702719

[20] Soliman S. and Bakulina A. , Hemophagocytic Lymphohistiocytosis After Inactivated Influenza Vaccination in a Young Man Complicated by Severe Rhabdomyolysis, Cureus. (March 2022) 10.7759/cureus.23334. PMC9018019 35475089

[21] Cattalini M. , Taddio A. , Bracaglia C. et al., Childhood Multisystem Inflammatory Syndrome Associated With COVID-19 (MIS-C): A Diagnostic and Treatment Guidance From the Rheumatology Study Group of the Italian Society of Pediatrics, Italian Journal of Pediatrics. (December 2021) 47, no. 1, 10.1186/s13052-021-00980-2. PMC7868856 33557873

[22] Gambadauro A. , Cucinotta U. , Galletta F. et al., A Case of Myocarditis in a Child With Multisystem Inflammatory Syndrome (MIS-C) Related to Previous Sars-CoV-2 Infection: Our Experience, Journal of Biological Regulators & Homeostatic Agents. (February 2022) 36, no. 1_Suppl_1.

[23] Pilania R. K. , Jindal A. K. , Bhattarai D. , Naganur S. H. , and Singh S. , Cardiovascular Involvement in Kawasaki Disease is Much More Than Mere Coronary Arteritis, Frontiers in Pediatrics. (September 2020) 8, 10.3389/fped.2020.526969. PMC7542237 33072669

