# Supplementary material for: H1N1‐Related Hemophagocytic Lymphohistiocytosis Complicated by Severe Rhabdomyolysis in a Pediatric Patient: A First Case Report
Source: Case Rep Pediatr. 2026 Mar 13;2026:3078496. doi: 10.1155/crpe/3078496 (PMC13674005; doi:10.1155/crpe/3078496)
Supplement: Supplementary file 1 [file CRPE-2026-3078496-s001.docx]

HLH-2004 diagnostic criteria. Adapted from Henter et al. "HLH-2004: Diagnostic and therapeutic guidelines for hemophagocytic lymphohistiocytosis" (2007)(5).

| Criterion | Description |
| --- | --- |
| 1. Fever | Core temperature ≥38.5°C |
| 2. Splenomegaly | Clinically detectable enlargement of the spleen |
| 3. Cytopenia | In ≥2 of 3 lineages in peripheral blood: |
|  | - Hemoglobin <9 g/dL |
|  | - Platelets <100,000/mm³ (or 10^9^/L) |
|  | - Neutrophils <1,000/mm³ (or 10^9^/L) |
| 4. Hypertriglyceridemia and/or Hypofibrinogenemia | - Fasting triglycerides ≥265 mg/dL (≥3.0 mmol/L) |
|  | - Fibrinogen ≤150 mg/dL |
| 5. Hemophagocytosis | In bone marrow, spleen, or lymph nodes (no evidence of malignancy required) |
| 6. Low or absent NK cell activity | Demonstrated by functional testing |
| 7. Ferritinemia | Ferritin ≥500 ng/mL |
| 8. Elevated soluble CD25 (sIL-2 receptor) | Soluble IL-2 receptor α ≥2,400 U/mL (value may vary slightly with lab standards) |

HLH is diagnosed if 5 out of the 8 criteria are fulfilled.

CD25=soluble interleukin-2 receptor α; NK=Natural Killer.
